# Zebrafish on a Chip: A Novel Platform for Real-Time Monitoring of Drug-Induced Developmental Toxicity

**DOI:** 10.1371/journal.pone.0094792

**Published:** 2014-04-14

**Authors:** Yinbao Li, Fan Yang, Zuanguang Chen, Lijuan Shi, Beibei Zhang, Jianbin Pan, Xinchun Li, Duanping Sun, Hongzhi Yang

**Affiliations:** 1 School of Pharmaceutical Sciences, Sun Yat-sen University, Guangzhou, China; 2 School of Pharmaceutical Sciences, Gannan Medical University, Ganzhou, JiangXi, China; 3 School of Laboratory Medcine, Hubei University of Chinese Medicine, Wuhan, China; 4 School of Pharmaceutical Sciences, Guangxi Medical University, Nanning, China; 5 The third Affiliated Hospital, Sun Yat-sen University, Guangzhou, China; East Carolina University, United States of America

## Abstract

Pharmaceutical safety testing requires a cheap, fast and highly efficient platform for real-time evaluation of drug toxicity and secondary effects. In this study, we have developed a microfluidic system for phenotype-based evaluation of toxic and teratogenic effects of drugs using zebrafish (*Danio rerio*) embryos and larvae as the model organism. The microfluidic chip is composed of two independent functional units, enabling the assessment of zebrafish embryos and larvae. Each unit consists of a fluidic concentration gradient generator and a row of seven culture chambers to accommodate zebrafish. To test the accuracy of this new chip platform, we examined the toxicity and teratogenicity of an anti-asthmatic agent-aminophylline (Apl) on 210 embryos and 210 larvae (10 individuals per chamber). The effect of Apl on zebrafish embryonic development was quantitatively assessed by recording a series of physiological indicators such as heart rate, survival rate, body length and hatch rate. Most importantly, a new index called clonic convulsion rate, combined with mortality was used to evaluate the toxicities of Apl on zebrafish larvae. We found that Apl can induce deformity and cardiovascular toxicity in both zebrafish embryos and larvae. This microdevice is a multiplexed testing apparatus that allows for the examination of indexes beyond toxicity and teratogenicity at the sub-organ and cellular levels and provides a potentially cost-effective and rapid pharmaceutical safety assessment tool.

## Introduction

Pharmaceuticals that may be administered to women of child-bearing age must be tested for their teratogenic and embryotoxic effects in rodent and non-rodent models [1]. In fact, whole animal models are valuable in drug safety testing because they provide correlative information that can be extrapolated to humans, and the embryos of such models are useful due to their high susceptibility to exogenous substances [2]. Recently, the embryos and larvae of zebrafish (*Danio rerio*), have emerged as a powerful and popular experimental model [3] for human disease and are used in drug discovery [4] and environmental toxicology [5].

Unlike mammalian embryos that grow inside their mother's uterus and make direct observation of embryonic development difficult [6], zebrafish embryos develop externally and their body is transparent enough to allow for characterization under a common optical microscope. Since it is a vertebrate, the zebrafish has tremendous potential as a genetic model due to its relatively short generation time (2–3 months), and high reproduction rate (over 200 progeny from one mother every week). More importantly, zebrafish develop similarly to mammals, including early embryonic processes and the development of the cardiovascular, somite, muscular, skeletal, and nervous systems [7]. In addition, recent studies have shown that zebrafish can be manipulated by genetic biotechnology for use in drug testing [8]. As a result, zebrafish embryos and larvae have been employed in organism-based high-throughput drug screening protocols due to their small size, low-cost, and compatibility with simple culture conditions [9].

In the majority of zebrafish-based pharmaceutical analysis and screening, the test platform mainly relies on multi-well plates. Thus, the embryos are exposed to the test drugs in culture solution over a specific length of time. Since the culture solution is not renewed, it evaporates and results in concentration and pH changes. Although one can fill several 96-well plates with embryos in one hour [10], the drug or culture solution still requires periodic renewal. However, frequent washing is time-consuming, and may injure the embryos. A modified 24-well plate with a flow-through system has been developed to alleviate this problem [11]. Unfortunately, this method has created another hurdle, since it requires a large volume of the test compound. Khoshmanesh established a microfluidic chip that docks zebrafish embryos automatically and cultures them under flow conditions [12]. However, this chip does not allow for the timely removal of the dead embryos which can theoretically influence the development of the remaining live embryos. Later, this same group designed a 3D multilayer microfluidic system for real-time developmental analysis of zebrafish embryos, but the design was ill-suited for fish egg exposure to multiple substances at different concentrations [13]. Recently, a multi-channel microfluidic perfusion platform was developed for culturing single zebrafish embryos and capturing live images of various tissues and organs inside the embryo [14]–[16]. Another approach for single embryo observation was developed by immobilizing individual eggs in microfabricated holes with culture solution flowing underneath [17]. Regrettably, both the chamber and the micro-hole are too small to manipulate dozens of embryos at one concentration. To address these issues, we fabricated an integrated microfluidic device composed of two parts [18]. One part of the device was composed of an open culture room array containing several embryos. Each culture room was simultaneously exposed to an upstream gradient of doxorubicin generated from another part of the chip. However, we found that the sloped chip design that facilitated the removal of waste did not allow for the real-time imaging of the fish embryo. Additionally, the glass based chip material was not biocompatible, to some extent, with live system that required gas exchange.

Herein, we describe a novel microfluidic device to simultaneously evaluate the developmental toxicity and teratogenic effect of Apl on zebrafish embryos and larvae using real-time imaging. The gradient stimulant was generated rapidly by a concentration gradient generator (CGG) microstructure, which was incorporated into a fish culture room array to support dose-dependent drug studies. The culture chamber has three repeated structure units and entraps the fish in sequence allowing for a reasonable statistical analysis. Upon continuous exposure to Apl, real-time imaging shows that it dose-dependently impairs the development of organs and tissues. Subtle changes in the transparent embryogenesis can be easily distinguished on a common optical microscope. The degree of fish injury was characterized by measuring the heart rate, survival rate, body length and hatched rate of the embryos. We believe that the microfluidic chip can serve as an excellent pharmaceutical evaluation platform for studying aminophylline-induced toxicity and teratogenicity during early embryonic and larvae development.

## Materials and Methods

### Materials

All chemicals and reagents were purchased from Sigma-Aldrich (USA) unless otherwise stated. A stock solution of 10 mg/ml of aminophylline (Apl) was prepared in E3 solution (5 mM NaCl, 0.17 mM CaCl_2_, and 0.16 mM MgSO_4_).

### Design and fabrication of the microfluidic device

**Figure 1:**
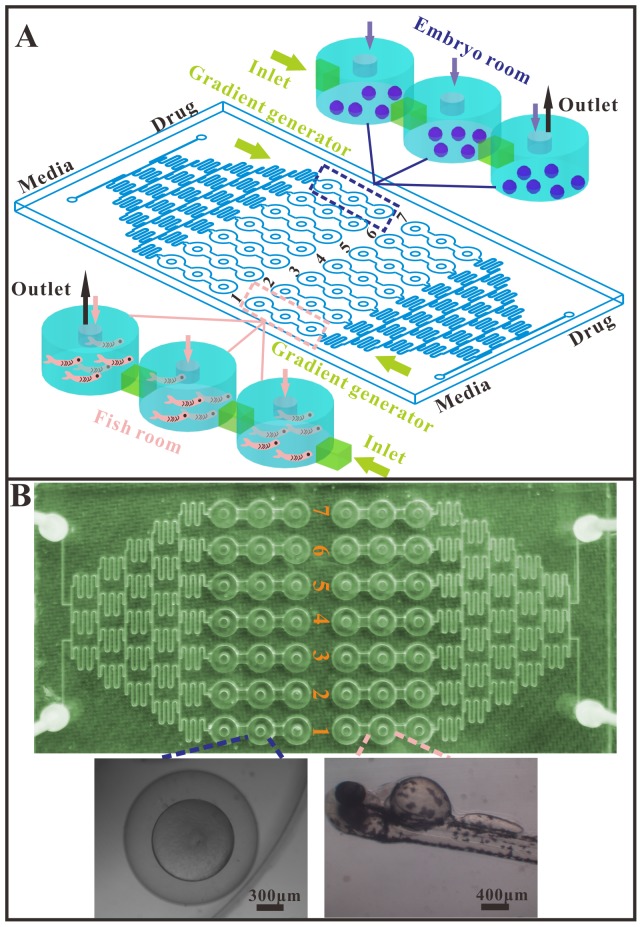
Embryonic and larvae zebrafish on microfluidic chip. (A) Schematic of chip for zebrafish assay. The chip includes two independent zones, each with a media inlet, a drug inlet, a gradient generator and seven series of fish tanks (one concentration with three tanks) named C1–C7. Left zone for embryonic toxicity and teratogenicity experiment, the right for larvae fish based drug toxicity evaluation. (B) Photos of the microfluidic chip and micrographs of the embryo and larvae in the chip.

The microfluidic chip (120 mm×60 mm, L×W) was composed of two independent units, and each unit contained a CGG, one media inlet, one drug inlet, 21 embryo inlets and outlets, 21 embryo chambers and 7 liquid outlets (Fig. 1). One unit was used for toxicity and teratogenicity experiments and the other unit was used for the larvae experiments. The drug was distributed by the CCG and was placed in chamber C1 (lowest concentration) to C7 (highest concentration).

The microchannel and culturing chambers were designed using AUTOCAD (2010) software (Autodesk, USA) and converted into a computerized numerical control (CNC) engraving and milling machine (Beijing, China) which has an accuracy of 30 µm. The channel patterns were then scribed into a copper mold (Fig. S1). The copper-based mold was used for molding the polydimethylsiloxane replica (PDMS, Sylgard 184; Dowcorning corp., USA). The PDMS elastomer base was mixed with curing agent at a 10∶1 (w/w) ratio, and degassed at 40 Torr to remove any residual air bubbles. The PDMS mixture was then poured onto the copper mold and cured at 65°C for 2 h. The PDMS wafer was allowed to cool to room temperature and then was peeled off the master mold. The chip was made of two layers. The top layer (3.5 mm thick) was made from the structured PDMS slice and contained two independent concentration gradient generators (CGGs), each with seven concentration exits that opened onto three linked embryo culturing chambers (6 mm in diameter, 2.5 mm in height). The bottom layer was made of glass (1.7 mm thick). The two layers were treated with an oxygen plasma generator (PDC-32G-2, Harrick, USA) and irreversibly bonded together at 80°C for 3 h.

The culturing medium and drug were delivered into the device by a syringe pump (LSP10-10B, LongerPump, Baoding, China), via Teflon tubes (ID 0.5 mm, OD 0.9 mm) connected to the inlets and outlets of the microfluidic chip. The liquid streams were designed to split, combine, and mix, and this process was repeated five times to generate seven concentration gradients distributed into the embryo culturing chambers. Then, the solutions were sent to the embryos or larvae fish trapped in the well-defined chambers.

### Flow simulation and characterization

In order to estimate the variations in velocity and pressure of the fluids and determine the hydrodynamic forces that may influence the embryonic development, computational fluidic dynamics (CFD) simulations of the flow pattern across the tanks were performed using Gambit 2.4 software (Fluent, Lebanon, NH, USA) to create the geometry and mesh generation. Fluent 6.3 software (Fluent, Lebanon, NH, USA) was used to simulate the flowing pattern in fish chamber. The gradient generator in this design had a sigmoidal distribution pattern, which was composed of a branching microfluidic network that created a concentration gradient by utilizing laminar flow and diffusive mixing [19]. When streams of various solutions flowed through the microfluidic network, they split at the nodes, combined with neighboring streams and were mixed in a 1∶1 ratio by diffusion. Theoretical concentration values were calculated based on the design of the sigmoidal concentration gradient generator. The Apl concentration in each fish tank on the chip was determined by an ultraviolet spectrophotometer (UV-3600, Shimadzu, Japan).

### Fish maintenance

The wild-type zebrafish (provided by School of Life Sciences, Sun Yat-sen University, China) were housed in a ratio of 2 females to 1 male in a stand-alone aquarium (L×B×H, 10 cm×20 cm×10 cm) with a flow-through system [20]. The fish were kept on a 14 h light/10 h dark cycle and the water temperature was set to 26±1°C [18]. Constant filtering or constant flow-through conditions ensured that ammonia, nitrite, and nitrate were maintained below detectable limits (0–5, 0.025–1 and 0–140 mg/L, respectively) [21]. The zebrafish were fed a commercially available, artificial diet (*Paramecium*) twice-a-day and overfeeding was strictly avoided to ensure optimal water quality. Uneaten food and feces were removed daily.

Female fish produce a large number of eggs and store them in a clearly recognizable protruding belly. Males and egg-laden females were housed separately but were placed in the same tank for one night during spawning. Then, zebrafish eggs were collected in a sedimentation tank using custom-built egg traps. The newly fertilized eggs were transferred into petri dishes filled with embryo medium E3. Note that the E3 solution did not contain methylene blue or antibiotics, and was rinsed to remove debris and dead embryos every 30 min [2]. Three hours post-fertilization (hpf) [22], the normally developing and healthy eggs were collected using a sterile micropipette with the aid of a stereomicroscope (SMZ-T4, Optec, Chongqing, China). The embryos were cleaned twice with E3 medium [23] and applied to the microfluidic chambers. The E3 medium was aerated for at least half an hour before the addition of the test chemicals [24].

### Continuous drug treatment of zebrafish embryos and larvae in microfluidic device

Freshly spawned eggs can be recognized by a fully transparent perivitelline space surrounded by the egg membrane, the yolk, and the germinal disc that forms at the animal pole. Only fertilized eggs were used for analysis and embryos with overt anomalies (asymmetries, formation of vesicles) or damaged membranes were discarded. Non-fertilized eggs can be identified by the lack of blastomeric formation and, at later stages, by their lack of transparency.

The microfluidic device was rinsed with 0.1 mol/l HNO_3_, 0.1 mol/l NaOH, ultrapure water and E3 solution, in that order. Then, the selected zebrafish embryos were transferred into the chamber by a modified pipette tip [18], and the embryos sedimented into the cylinder-shaped chambers. For the microfluidic chip embryo toxicity test, 30 freshly zebrafish eggs (3 hpf) were selected and transferred into three chambers so that there were 30 embryos tested for each concentration. To facilitate the trapping process, each culture compartment was prefilled with culture medium. The waste solution was discharged from the liquid outlets. In this experiment, some of the embryos were cultured with fresh culture medium while others were cultured in medium contained 10 mg/ml Apl (Sigma Chemicals, St. Louis, MO). The flow stream was created by a syringe pump with positive pressure to a flow rate of 5 µl/min and the microchip was maintained in a custom-designed incubator at 28.5°C to ensure the normal development of the embryos. After a 24 h treatment (the zebrafish embryos are in pharyngula period), the test chemicals were replaced by the E3 solution, and the embryos and the larvae were observed directly under a microscope (SMZ-T4, Optec, Chongqing, China). In the larvae-based experiment, the two inlets were perfused with fresh medium until the embryos hatched which normally required 72 h. The drug (10 mg/ml Apl) was added through the drug inlet at a flow rate of 5 µl/min after all of the embryos hatched. It is important to note that the chip has two independent zones to allow for the zebrafish embryo and larvae experiments to be conducted at the same time. All of the experimental procedures were carried out in accordance with the China Animal Welfare Legislation, and were approved by the ethics committee on the Care and Use of Laboratory Animals at Sun Yat-sen University, Guangzhou, China.

### Assessment of toxicity, teratogenicity and endpoint

Several toxic and teratogenic effects were evaluated, including the developmental toxicity of stage-specific and continuous treatment and the teratogenic effects of treatment at 3 to 96 hpf in embryonic zebrafish. The chip networks provided a means for the creation of an Apl concentration gradient to determine its toxicity and teratogenicity. Throughout the entire exposure period post-fertilization, the embryos and larvae were observed under an inverse microscope (Olympus IX51, Tokyo, Japan) and their responses were documented at specified time points (t = 12, 24, 36, 48, 60, 72, 84, and 96 h).

All embryos were staged as described by Kimmel et. al. [25]. During the experiment, the assessment of terminal end points were recorded, including the lack of a heartbeat, blood circulation and motility, which together characterize a lethal or teratogenic effect according to Nagel [26] and Bachmann [27].

Teratogenic effects were considered to be finger print endpoints if the following criteria were fulfilled: (i) concentration-response relationship and (ii) the endpoint must be observed in ≥50% of all embryos [23]. In addition to these finger print end points, the convulsion of zebrafish was recognized as intense body tics, with the number of convulsionary fish being directly calculated by optical microscopy.

### Image and LC_50_ analysis

An inverted optical microscope (4×, 10× objective, BDS200, Optec, Chongqing, China) with a digital camera (DM200) was used for a visual toxic impact of the test chemicals on the embryonic development. The evaluating endpoints were heart rate, body twisting, pigmentation, tail detachment, development of eyes, segmentation, and teratogenicity. The recorded photographs were analyzed using OPTPRO software. A 10× objective was used to get comprehensive information on the target feature of the deformations caused by the chemical treatment. The quantitative endpoints, spontaneous movements and heart rate were measured for 20 s at 24, 48, 72 and 96 hpf, respectively.

The calculation of LC_50_ values was determined by the software SPSS (IBM, Statistical Product and Service Solutions, Version 11.0) with the data being processed using probit analysis for linear maximum likelihood regression or moving average computation.

### Statistical analysis

The data presented in this study was checked for normality and homogeneity of variances by the Kolmogorov-Smirnov one-sample test and the Levene test. The differences between groups were calculated with a one-way analysis of variance (ANOVA) followed by a Dunnett's multiple comparison test using the SPSS 11.0 software ((IBM, Statistical Product and Service Solutions, Version 11.0) with a p value of *p*<0.05 considered to be statistically significant. The figures were drawn using Origin 6.0 (OriginLab, Northampton, MA, USA).

## Results and Discussion

### Chip design and flow characterization

**Figure 2:**
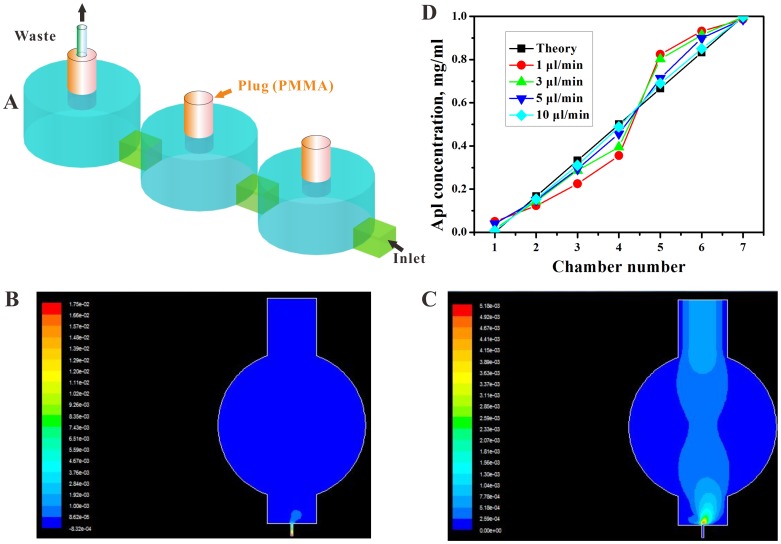
Schematics of liquid direction, the cylinder plug and concentration distribution by CGG. (A) The first chamber with a liquid inlet and cylinder plug, the second chamber with a cylinder plug only, the last chamber with a hollow cylinder lid for liquid out. (B) Simulation of the pressure pattern at the first chamber at flow rate of 0.01 m/s. (C) Simulation of the velocity pattern at the first chamber at flow rate of 0.01 m/s. (D) The average gradient concentration (n = 3) of 1 mg/ml Apl by CGG were quantified at different velocities.

A microfluidic chip consisting of two layers (top PDMS layer, bottom glass layer) was fabricated by utilizing the integration of an array of embryo trapping chambers with a concentration gradient generator. In this device, a newly designed chamber configuration of three repeated consecutive units causes the entrapment and culture of zebrafish embryos or larvae. By stopping the transfer of embryos into the wells using a modified pipette tip, a transparent PMMA cylinder plug (Ф2 mm×5 mm) was inserted to seal the fish inlet, while keeping the end chamber with a hollow plug connected to a Teflon tube for removal of waste [Fig. 2A]. This consecutive open design can remove the dead embryo easily and enables a precise and continuous real-time analysis. Furthermore, a relatively large micro-channel (2 mm wide) connection between two chambers was applied to reduce the flow rate and thus eliminate any chance of harmful fluidic shear stress (Fig. 2B and Fig. 2C). By the end of the drug treatment, a flow velocity as high as 50 µl/min of E3 solution was used to remove the remaining drug(s) for interference-free imaging at the single organism level. This rapid elution process was characterized by the use of a diluted methylene solution.

Another important feature of this chip was the CGG in the chip. The theory and equation of calculating the distributed concentration of dissolved chemicals using the CGG has been described by Joen *et.al.* [28]. Based on their theory, 1 mg/ml of an Apl solution at different velocities combined with the CGG apparatus can be used to rapidly generate a multiple concentration gradient. The collected drug concentrations were determined by UV absorption at 275 nm (Fig. 2D). The data demonstrated that the CGG generates concentrations with a high degree of accuracy when the velocity of the solution is less than 5 µl/min. While the speed is above 5 µl/min, the drug concentration profiles are close to the theoretical value, and this may be due to the high flow rate that reduces the laminar flow of the solution.

### Embryos development on chips

To develop a reliable and precise drug evaluation microdevice for zebrafish, we first examined the development and growth of an embryo within a chip culture chamber by exposing it to flow stress. The embryos at 3 hpf were dispensed into the culture rooms (10 eggs per room, namely 30 eggs per concentration). Multiple flow rates ranging from 0 µl/min to 10 µl/min were examined for their effects on the development of the embryos. In addition, the zebrafish (3 hpf) were cultured in a 24-well plate (10 per well) with periodic renewal of E3 buffer solution every 24 h as a control. Note that the hatching rate and body length of the whole fish are two critical indicators for estimating the developmental state of the zebrafish.

**Figure 3:**
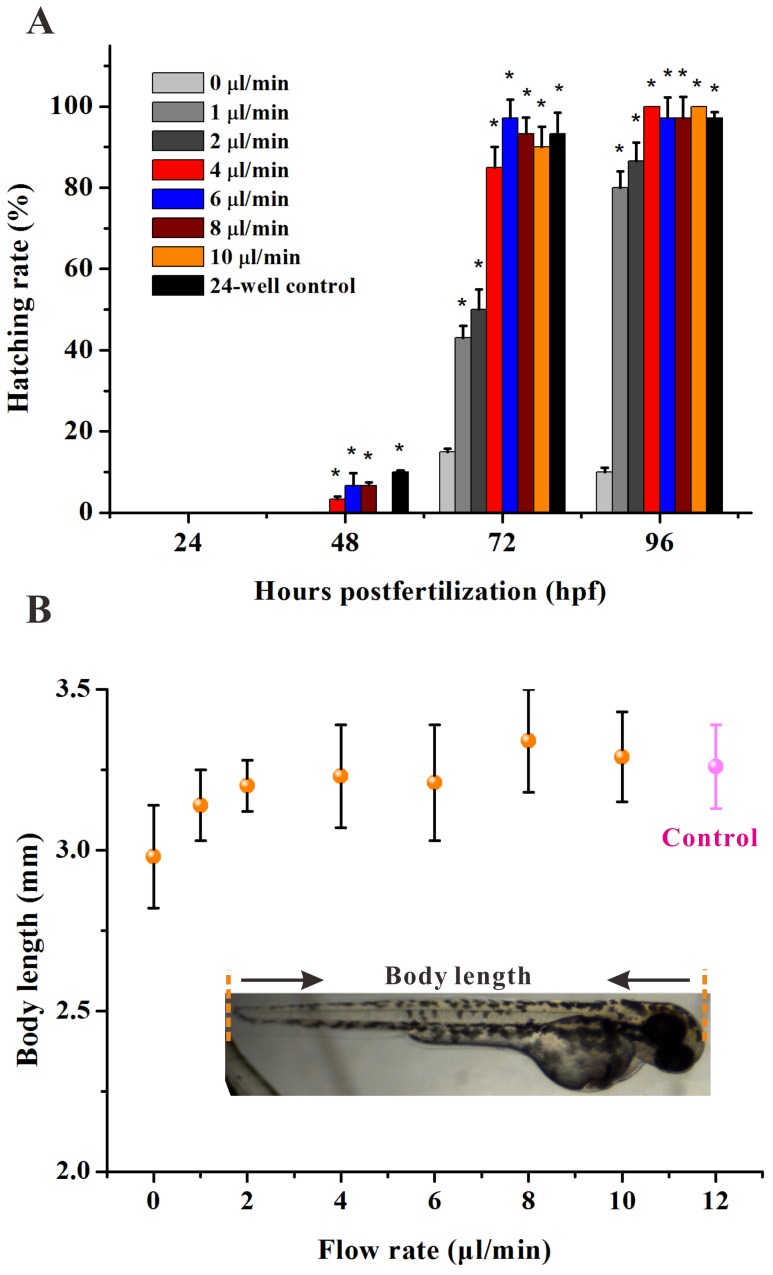
Development of Zebrafish embryos in microfluidic chip under flowing stream. (A) Hatching percentage of embryos cultured in the chip by continuous exposure at varied flow rates and at different growth periods. The asterisks indicate significant differences from control group (chamber 1) * at *p*<0.05. (B) The body length of the larvae fish at 96 hpf (n = 20) cultured in the chip by continuous exposure at varied flow rates (pink control is body length of the larvae fish cultured in 24 microwells).

During the 4 day culture period, a time-dependent imaging analysis of the fish body and organs was performed to examine the developmental stages at every 24 hpf point. It was observed that most of the embryos hatched successfully with full-grown organs and the newborn larvae had a complex behavior. Both the static micro-well with a periodic E3 renewal and microscale flow-through platform exhibited high hatch rates but the flow rate at 0 µl/min caused a hatching rate of less than 20% (Fig. 3A). Moreover, the low flow rates below 4 µl/min in the chip tended to delay or suppress hatching when compared to the control chambers where the hatching rate reached up to 97.1% at 72 hpf. The results showed there was a minimal impact on the embryonic development when the solution velocity was higher than 4 µl/min in the microdevice.

Estimation of the growth of the embryo was based on the body length of the grown larvae fish. At 96 hpf, an imaging measurement was obtained showing the shortest length of the fish at 0 µl/min flow rate (Fig. 3B) and the length of the juvenile body exceeded 2.8 mm at each flow rate, suggesting that the oxygen in the chip is sufficient for embryonic development.

### Toxicity and teratogenicity of Apl on zebrafish embryo and larvae fish

As a whole-organism model, the zebrafish embryo has had a broad utility in the toxicity and teratology fields for decades [29]. Teratogenicity assays *in vitro* have become increasingly significant for supporting the “3R principle” (reduction, refinement and replacement of animal use). Apl is mainly used to treat bronchial asthma, bronchitis, emphysema but can also be used for cardiogenic pulmonary edema. However, Apl has side effects causing a range of toxicity such as cardiotoxicity [30] and convulsions [31]. The acute embryotoxicity of Apl exhibited an obvious time and dose dependent toxicity and the LC_50_ value (Lethal Concentration 50) to the zebrafish embryo was 5.0 mg/ml in a microwell at 84 hpf. Our group used Apl to examine its developmental toxicity and teratogenicity on zebrafish embryo and larva. Fig. S2 shows the hatching rates of the zebrafish embryos in 24-well plates and the LC_50_ was shown to be at 5.4 mg/ml at 84 hpf.

### 1. Developmental retardation, mortality and abnormal morphology

**Figure 4:**
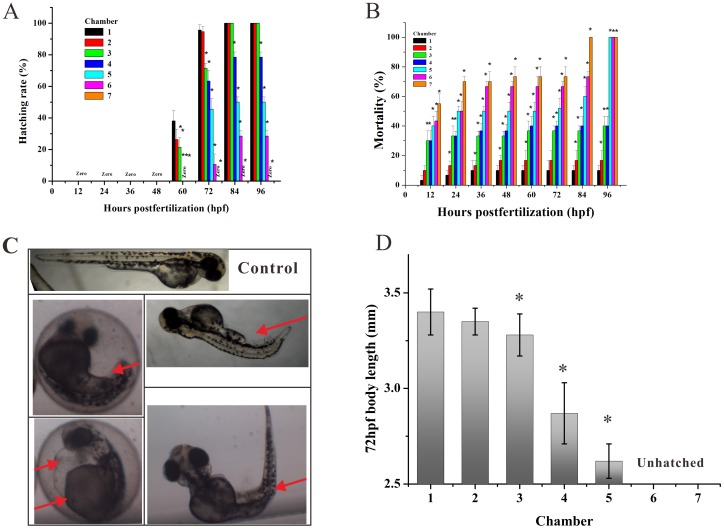
Apl induced abnormal morphology, developmental retardation, and mortality of zebrafish embryos. (A) Hatched rate and (B) mortality of zebrafish embryos exposed to gradient Apl every 12 hpf in the microfluidic chip for 96 h. (C) Typical morphological abnormalities of embryos exposed to Apl. Red arrows indicate tail malformation, delayed yolk absorption, pericardial edema, and bent trunk, respectively (from upper left-right to bottom left-right). (D) Mean body length of hatched embryos treated with Apl in the chip compared with the controls (C1) at 72 hpf (n = 6), there are no date at C6 and C7 for the fish are not hatched. The asterisks indicate significant differences from control group (chamber 1) * at *p*<0.05.

During the periods of rapid embryogenesis, seven representative developmental stages (zygotic cell, cleavage, blastula, gastrula, segmentation, pharyngula, and hatching period) have been defined to allow subsequent assimilation of new observations and details. This has resulted in the investigation related to embryonic development [25]. After exposure to gradient Apl from 3 hpf to 96 hpf, our group observed slight developmental retardation during early embryogenesis (before 48 hpf) and the treated embryos appeared head-trunk angle compared with the controls (Fig. 4A). Thus, it was found that developmental retardation at high concentrations of Apl, such as C5, C6 and C7 groups exhibited lower hatching rates at 72 hpf (Fig. 4A). These results indicate that when the concentration of Apl is higher than C2, it may actually inhibit the normal development of the zebrafish embryo.

As showed in Fig 4B, embryo mortality was clearly dose-dependent and the mortality level increased as the concentration of Apl administered increased. At 3 to 84 hpf, exposure of the lowest concentration of Apl (C1) had no effect on survival rate, and the mortality was very close to that of the control group at 96 hpf. However, the mortality increased sharply at the C5 concentration and almost 50% of the embryos died at 12 hpf (Fig.4B).

Another important parameter to evaluate the teratogenicity of a drug on zebrafish is abnormal morphology. During exposure, embryos in the C1 group had a normal yolk absorption with a long straight body and a normal spinal cord (Fig. 4C). Nevertheless, the treated embryos from C2 to C7 groups exhibited a consistent and highly reproducible pattern of developmental abnormalities including delayed development, pericardial edema, bent trunk, skeletal malformations, tail malformation and delayed yolk absorption (Fig. 4C). Furthermore, malformations appeared earlier during development with increasing Apl concentration. Interestingly, the malformations caused by low concentrations of Apl would gradually become pronounced during subsequent days of larval development with the indication that the injury is irreversible. The toxicity and teratogenicity of Apl was additionally supported by a significant reduction of body length at 72 hpf in the groups exposed to a high drug concentration (Fig. 4D). This data shows that the concentration surpassing C4 group would result in a damaged spinal cord of hatched fish.

### 2. Cardiac abnormal development

**Figure 5:**
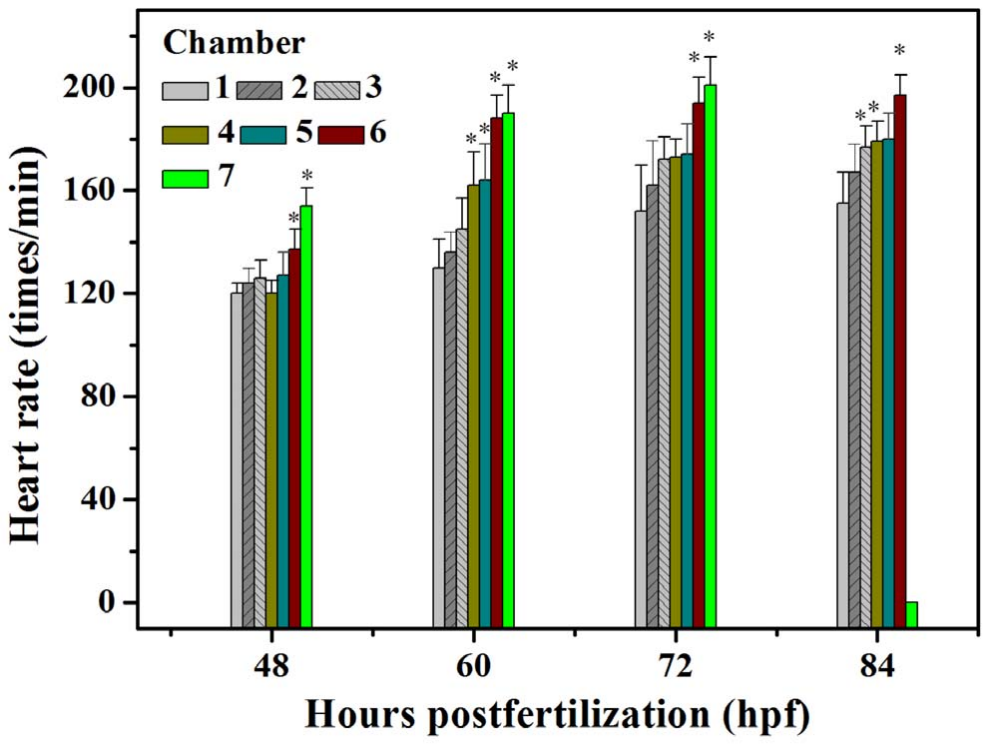
The heart rates of treated embryos from 48 to 84 hpf by measuring every 12 hpf, the heart rate of the embryos in chamber 7 is zero at 84 hpf. The asterisks indicate significant differences from control group (chamber 1) * at *p*<0.05.

It has been reported that the heart is first visible at approximately 24 hpf as a cone-shaped tube behind the brain. At approximately 48 hpf, a bend in the heart marks the division between the atrium and ventricle and then the heart tube is bent to bring the atrium to a position dorsal to the ventricle at 60 hpf [32], [33]. The heart rate is used as an index of the effect of Apl exposure on cardiac development. The heartbeat of the embryos gradually increased between 48 and 84 hpf between the C1 control and other drug treated groups but it was noted that the higher concentrations of Apl resulted in a faster heartbeat (Fig. 5). Both Apl and flumenthrin increased the embryonic heart rate, but toltrazuril decreased it [34]. More significantly, bradycardia was observed in the treated embryos at 72 to 84 hpf in the C7 group, which confirmed the embryonic toxicity of Apl (Fig. 5). Pericardial edema, an important signal in embryonic cardiac developmental toxicity was also observed in Apl treated embryos. This impairment began to be measurable at 36 hpf, particularly for the embryos in chamber 5, 6 and 7.

### 3. Convulsion and death of zebrafish

In effect, the embryos had tail tremble during embryogenesis after 48 hpf with exposure to high concentrations of drug (in C4–C7). Furthermore, the hatched larvae also appeared to have tremors and convulsions (movie S1). Thus, we defined the term ‘clonic convulsion rate’ as the percentage of convulsionary fish appearing in the three interconnected chambers. The convulsion frequency was positively correlated to impairing severity, which was clearly identified as a time and concentration dependent increase at 96 hpf.

**Figure 6:**
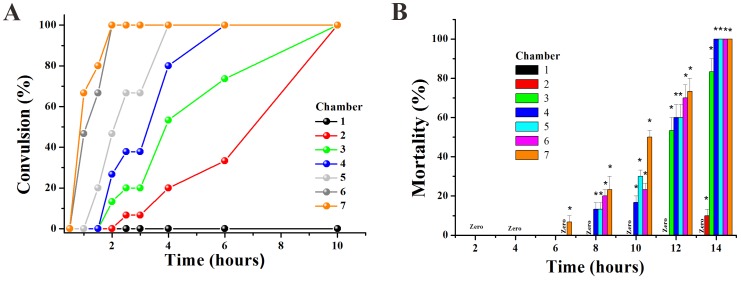
Apl induced convulsion and mortality of larvae zebrafish aged 72 hpf. The convulsion rate (A) and mortality (B) of larvae fish after treatment by gradient Apl. The asterisks indicate significant differences from control group (chamber 1) * at *p*<0.05.

In order to identify the toxicity effect of Apl, we exposed 72 hpf hatched larva to a concentration gradient of Apl. We found that fish in the high concentration Apl such as C6 and C7 groups exhibited convulsion after a 1 hour treatment with Apl, and this effect was time and concentration-dependent (Fig 6B). The higher concentration (C6, 8.99 mg/ml or C7, 9.97 mg/ml) of fish showed tics within a shorter period of time. More importantly, all larvae demonstrated convulsions after 10 hours in the presence of Apl (Fig. 6A).

The death of larvae in the C7 group occurred 6 hours after treatment with Apl, and larvae in the C4, C5, C6 and C7 groups died after 14 hours of treatment (Fig. 6B). The larvae died from heart bleeding suggesting that Apl is toxic to the cardiovascular system.

## Conclusions

In this study, we developed a microfluidic system to evaluate the dynamic developmental toxicity and teratogenicity of clinical drugs on both zebrafish embryos and larvae simultaneously. The microfluidic device offers two independent functional units for embryo and larvae exposure experiments, and each unit includes a gradient concentration generator, egg chamber array, drug/E3 solution inlets and waste outlets. The embryo can develop normally in microscale tanks with open structures to remove dead embryos quickly. Dynamic development in the presence or absence of Apl can be easily characterized by common optical imaging in a real-time manner. Apl causes dose- and time-dependent embryonic toxicity and teratogenicity. This was shown as delayed development, pericardial edema, bent trunk, skeletal and tail malformations. Convulsion was another important indicator used to assess the side effects of the drug on embryo and larva zebrafish. This innovative microsystem has great potential to compete with a microplate platform to perform pharmaceutical safety evaluations and drug screening using embryonic or young zebrafish as vertebrate models, especially at the single organism level [35].

## Supporting Information

Figure S1 **Schematic of an integrated microfluidic chip for zebrafish-based drug toxicity assay.** (A) The CAD design picture before the copper-formed Mold. (B) Copper-formed Mold for the microfluidic. (C) The top-layer made from PDMS. (D) The bottom layer is plate glass. (E) A two-layer microfluidic chip. (F) Magnified section of one concentration connecting three chambers for zebrafish embryos experiment.
(TIF)

Figure S2 **Development toxicity of Zebrafish embryos in 24-wells plate.** (A) Hatched rate and (B) mortality of zebrafish embryos exposed to Apl every 12 hpf in 24-wells plate at the same concentration generated by the CGG.
(TIF)

Movie S1 **Apl-induced rhythmic tremble and rotation in high frequency for zebrafish larvae.**
(AVI)
