# Supplementary figures and images for: Zebrafish on a Chip: A Novel Platform for Real-Time Monitoring of Drug-Induced Developmental Toxicity
Source: PLoS One. 2014 Apr 14;9(4):e94792. doi: 10.1371/journal.pone.0094792 (PMC3986246; doi:10.1371/journal.pone.0094792)

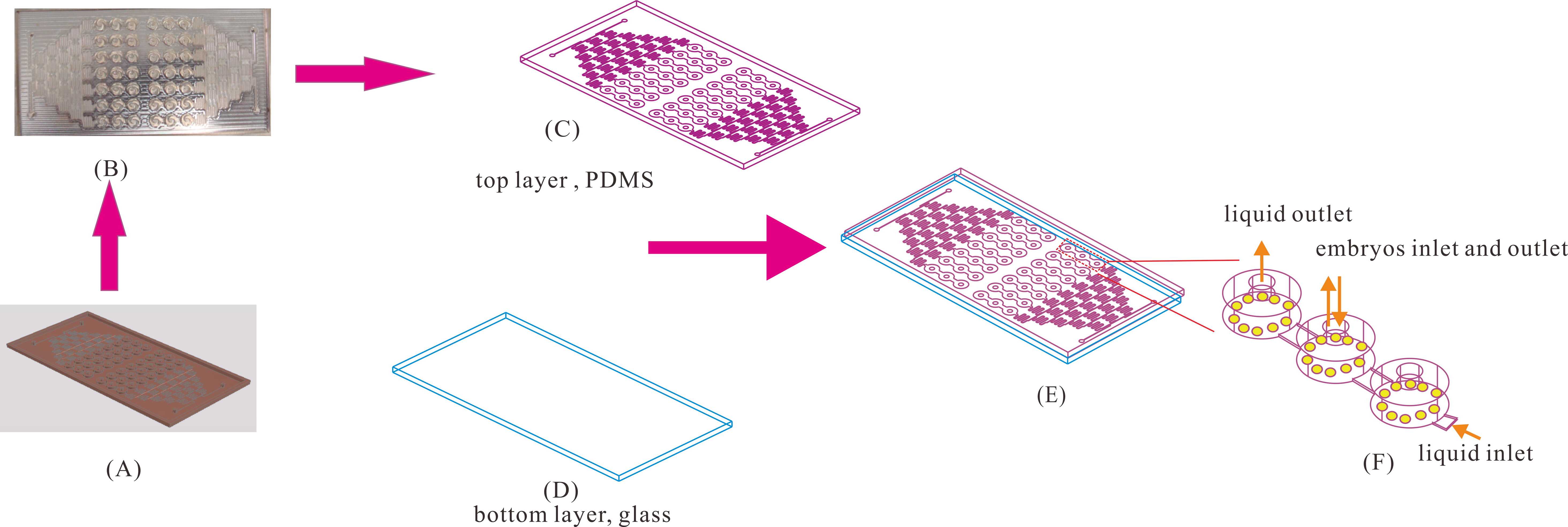

Supplement: Figure S1 — Schematic of an integrated microfluidic chip for zebrafish-based drug toxicity assay. (A) The CAD design picture before the copper-formed Mold. (B) Copper-formed Mold for the microfluidic. (C) The top-layer made from PDMS. (D) The bottom layer is plate glass. (E) A two-layer microfluidic chip. (F) Magnified section of one concentration connecting three chambers for zebrafish embryos experiment. (TIF) [file pone.0094792.s001.tif]

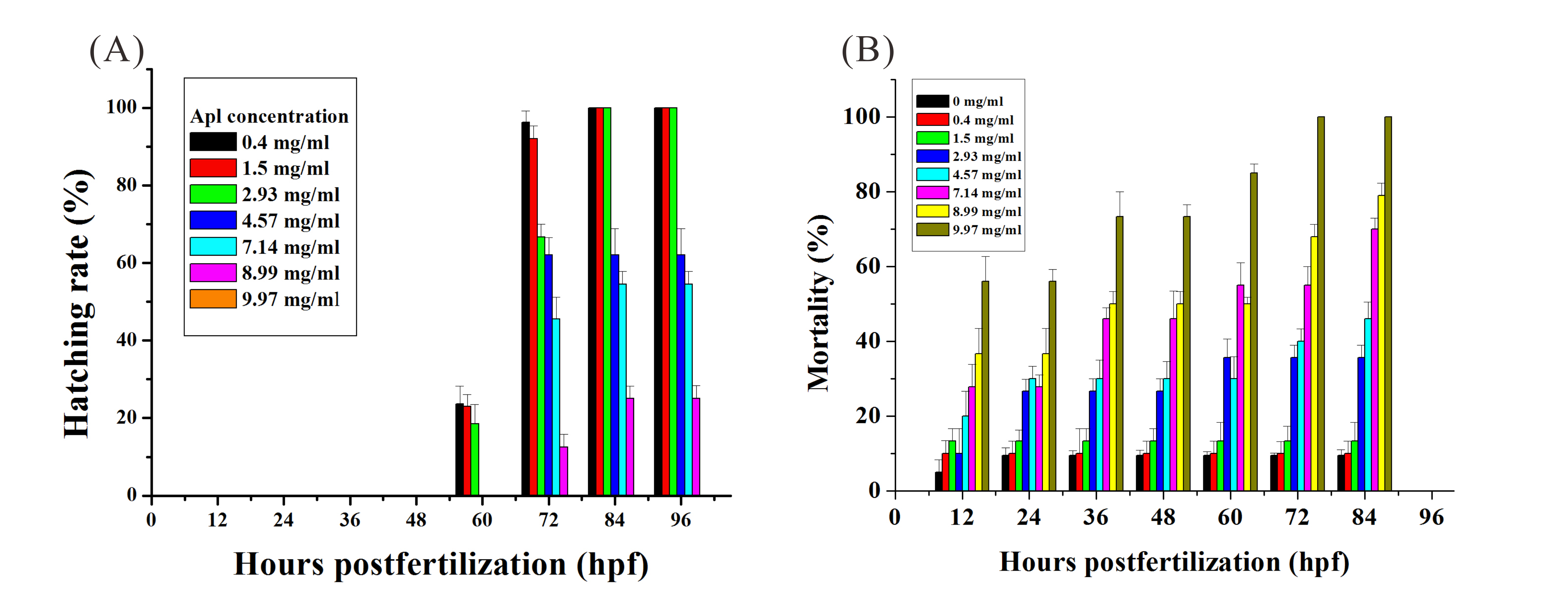

Supplement: Figure S2 — Development toxicity of Zebrafish embryos in 24-wells plate. (A) Hatched rate and (B) mortality of zebrafish embryos exposed to Apl every 12 hpf in 24-wells plate at the same concentration generated by the CGG. (TIF) [file pone.0094792.s002.tif]
